# The contribution of neurocognitive situation, physical capacity and daily life activities to quality of life in childhood acute lymphoblastic leukemia survivors

**DOI:** 10.3906/sag-2010-299

**Published:** 2021-10-21

**Authors:** Elif KELEŞ GÜLNERMAN, Yağmur ÇAM, Bülent ELBASAN, Şebnem SOYSAL, Zühre KAYA, İdil YENİCESU, Ülker KOÇAK

**Affiliations:** 1 Department of Pediatrics, Faculty of Medicine, Gazi University, Ankara Turkey; 2 Department of Physiotherapy and Rehabilitation, Faculty of Health Sciences, Hatay Mustafa Kemal University, Hatay Turkey; 3 Department of Physiotherapy and Rehabilitation, Faculty of Health Sciences, Gazi University, Ankara Turkey; 4 Division of Pediatric Hematology, Department of Pediatrics, Faculty of Medicine, Gazi University, Ankara Turkey

**Keywords:** ALL, QL, neurocognitive and physical outcome

## Abstract

**Background/aim:**

There are no extensive studies on the QL in children who completed acute lymphoblastic leukemia (ALL) treatment and currently living without any disease in Turkey. Our study aimed to analyze both the QL and the effects of physical, neurocognitive capacities on QL in childhood ALL survivors aged 7–12 years at the time of recruitment.

**Materials and methods:**

PedsQL cancer module 3.0 child and proxy report, for ages 5–7 and 8–12 years, WeeFIM scale, BOTMP Short Form, RPM, reading, writing, and mathematics assessment tools, sociodemographic information form were carried out to the children and their family.

**Results:**

There was no effect of the months since the completion of therapy on pain, anxiety, cognitive problems, perceived physical appearance, and the total QL scores of children and proxy reports (p > 0.05).Children’s physical capacities were significantly worse than healthy controls and have not reached the level of healthy children even after a long time since completion of ALL therapy. There was a significant association between physical capacity and daily independent living status (p < 0.001). Reading, writing, and mathematical skills were significantly associated with the mean time off-treatment (p < 0.001), and the total score of RPM and PedsQL of those with mathematical difficulties were significantly lower than those without any difficulty (p < 0.05).

**Conclusion:**

The months after the treatment (off-treatment time) have not affected total and subunit QL scores. As motor skills difficulties will lead to low academic achievement, early recognition direct the parents for immediate intervention. lead to low academic achievement, early recognition could direct the parents for immediate intervention. Planning psychosocial support programs for physical activity and age-appropriate development of patients from the initiation of treatment will increase the QL in childhood ALL with a survival rate of 80% or more.

## 1. Introduction

ALL is the most common cancer type of childhood and accounts for 26.8% of childhood cancers worldwide [1]. Increased success in treatment and survival rates in children with ALL leads to decreased short and long-term morbidity, which could be assessed by measuring QL [2]. The QL is defined as the perception of the situation in life in terms of its own goals, expectations, and concerns with respect to the cultural structure and values system in the patient’s lives. Health-related QL is a multidimensional concept including the wellbeing of the patient in terms of physical, emotional, mental, and social behaviors [2].

With modern treatment methods, the overall life expectancy in childhood cancer has increased to 80%, and a chronic process starts after treatment despite full recovery [3,4].

There are no extensive studies on the QL in children treated for ALL and currently living without disease in Turkey. Our study aimed to analyze both the QL and the effects of physical, neurocognitive capacities on QL in pediatric ALL survivors aged between 7–12 years at the recruitment time.

## 2. Materials and methods

Thirty-five patients (33 pre-B ALL, 2 T-ALL) between the ages of 7–12 years currently have no disease after ALL treatment, and their families agreed to participate in our study. All patients were treated same chemotherapy protocol (ALL-BFM 2000) and not physically or mentally impaired at the study time. The mean age at diagnosis was the same in all age groups currently completed treatment; treatment protocols and risk stratification according to the age varied no difference between the age groups. None of the patients had CNS involvement at the time of diagnosis. Informed consent and basic demographic information by face to face interview, PedsQL 3.0 child and proxy report, for ages 5–7 years and 8–12 years [5], WeeFIM scale [6], BOTMP Short Form (BOTMP-SF) [7], RPM, reading, writing, mathematics assessment tool [8,9] were carried out children and their families (Supplementary material).

ALL is more common between 2–5 years of age, rendering a dynamic age group in whom psychological and social development might be disturbed by this life-threatening disease. The selection of patients within 7–12 years of age is since children who completed ALL therapy were thought to assess returning to school and social life. Regular interventional procedures, hospital visits, school interruptions, and reduced social activities all lead to conflict. All these factors hamper psychological and social development and affect future academic life [4]. Since the “QL scale” specific to cancer itself was used rather than the generic scale, no control group, including healthy children, was established.

Patients with a neurocognitive problem lead to physical disability or immobility, and any previous psychiatric diagnosis, as well as those who were refusing to participate in the study, were excluded. Statistical analysis was performed using the SPSS v. 20.0 program. The normal distribution of the variables was analyzed by Kolmogorov–Smirnov and Shapiro–Wilk tests. The Mann–Whitney U test was used for the comparison of pairs. One sample t-test was used to compare the data from the expected value and the study. The independent t-test was used to evaluate physical capacity tests. Pearson correlation analysis was used for the data with normal distribution, and Spearman correlation analysis was used for data without normal distribution.

## 3. Results

The demographic characteristics of the patients are shown in Table 1. The mean age of the study group was 9.3 ± 1.8 years. The age of diagnosis was 4.6 ± 2.1 years. Mean time off-treatment was 49.1 ± 30.9 months (1.4–125 months). Because the mean time after the end of the therapy (off–treatment) was 49.1 ± 30.9 months in our patients, the PedsQL child and proxy report total and subunit scores were compared according to 49 months. The subunit scores of QL significantly improved after 49 or more months since the end of the therapy, has been shown in Table 2. There was no significant improvement in the scores of pain, anxiety, cognitive problems, perceived physical appearance, and the total QL scores in survivors (p > 0.05) (Table 2). PedsQL child and proxy report total scores were significant and positively correlated (p < 0.001). PedsQL child score subunits like procedural anxiety (p < 0.05), treatment anxiety (p < 0.05), cognitive problems (p < 0.05), and perceived physical appearance score (p < 0.05) were positively correlated with the corresponding PedsQL proxy subunits.

**Table 1:** Sociodemographic characteristics according to patients’ ages.

Age of patients	(n)	Age at diagnosis	
		Mean ± SD	Median(min-max)
7 years	6	4 ± 1.4	4(2–6)
8 years	9	4.2 ± 1.7	4(2–7)
9 years	4	5 ± 1.4	4.5(4–7)
10 years	5	4.2 ± 1.9	4(2–7)
11 years	4	4.5 ± 2	4.5(2–7)
12 years	7	5.8 ± 3.3	6(2–10)
Educational status	(n)		
Not going school	4		
1st year	7		
2nd year	6		
3rd year	6		
4th year	3		
5th year	9		

**Table 2:** Effect of off-treatment time on the quality of life.

	PedsQLchild reportnausea	PedsQLchild reporttreatment anxiety	PedsQLchild report communication	PedsQL proxy report procedural anxiety	PedsQL proxy report treatment anxiety
2–48 months (n: 20)	77.7 ± 18.4	72.9 ± 31.2	63.3 ± 28.7	50 ± 36.9	51.2 ± 44
≥49 months (n: 15)	81.7 ± 17.6	95 ± 10.8	83.9 ± 28.1	78.9 ± 27.4	86.7 ± 17.5
p	p < 0.05	p < 0.05	p < 0.05	p < 0.05	p < 0.05

There was a significant difference in the perceived physical appearance between the scores of male patients’ parents and the female patients’ parents (p < 0.05). There was no significant association between months after the end of the therapy and the effect of QL and subunits of children and proxy reports (p > 0.05). The off-treatment time was significantly associated with WeeFIM total score, transfers, and social status subscales (p < 0.05). Wee-FIM score was not associated with parental education level and family income (p > 0.05). There were significant associations between WeeFIM and PedsQL child (p < 0.01) and proxy reports (p < 0.01).

BOTMP SF total score of healthy children was significantly different from the pediatric ALL survivors (p < 0.001). BOTMP SF total score was significantly associated with only two dimensions regarding gross motor skills [“tapping feet alternately while making circles with fingers” (p < 0.05) and “throwing a ball at a target with the preferred hand”]. The subunit scores of fine motor skills [“speed response” (p < 0.05), “drawing a line through a straight path with the preferred hand” (p < 0.001), “copying a circle with the preferred hand” (p < 0.05), “sorting shape cards with preferred hands” (p < 0.001) “making dots in circles with preferred hand point by circle” p < 0.001)] in pediatric ALL survivors were lower and significantly different from the healthy children’s scores.

There was a significant difference between the BOTMP SF mean total score of 20 children whose off -treatment time was ≤ 49 months than the BOTMP SF mean total score of 15 children whose off-treatment time >49 months (p < 0.001). There was a negative correlation between the age of diagnosis and the total BOTMP SF score (p < 0.05). The off-treatment time was significantly associated only with five dimensions out of 14 (running speed and agility, standing broad jump, catching a tossed ball with both hands, walking forward on the balance beam, and speed response). The BOTMP SF score was not significantly associated with the parental education level and family income (p > 0.05).

The neurocognitive functions of our patients were within normal limits. RPM scores of the female patients in the first, second, and fifth grades of the school were higher and significantly different from the healthy control scores (p < 0.05). 

Forty percent of patients had writing and mathematical difficulties, and 60% had reading difficulties. The mathematics score was significantly associated with parental education level and family income (p < 0.05). RPM score was significantly associated with the score of mathematics (p < 0.01), reading (p < 0.01), and writing skills (p < 0.05). There was a correlation between the off-treatment time and reading (p < 0.001) and mathematics skills (p < 0.001). There was a significant difference between the mean scores of PedsQL child and proxy reports and RPM scores of children with mathematical difficulties than those without mathematical problems (p < 0.05). There was no significant difference regarding fine motor skills between the children with difficulties in reading, writing, and mathematics compared to those without any problems (p > 0.05).

## 4. Discussion

There are no studies in our country focused on disease-specific QL in childhood ALL survivors. ALL patients aged 13–18 years were reported to have QL that did not differ according to age, sex, monthly income, parental education level on the generic QL scale [10]. Another study from our country studied 70 ALL survivors aged between 7–17 who were off therapy ≥ 2 years. No differences were found among all survivors’ QL subscale scores, including sex, therapy type, risk group, time after treatment, income status, chronic illness, and relapse history [11]. Our study also showed no differences in the QL according to age, sex, parental education, socioeconomic level, and off-treatment time, similar to the other Turkish studies [10,11] and a study from Indonesia [12]. Sung et al. showed that the QL of children on treatment is worse than off treatment for ≥12 months; however, there was no difference in the patients’ QL before and after the maintenance therapy [13]. A prospective study by Vlachioti et al. in 56 patients between 7–20 years with ALL and other childhood cancers showed that QL did not change during the treatment [14]. 

There was no effect of the off-treatment time on pain, anxiety, cognitive problems, perceived physical appearance, and the total QL scores of children and proxy reports in our study. However, other prospective studies showed an increase in the QL of patients with ALL at the end of therapy [15,16]. 

Compared to the results of a Canadian study by Sung et al., higher scores in the subunits of pain, nausea, and procedural and treatment anxieties in our study indicate better QL [13]. This could be because the off-treatment time was longer (≥49 months) in our study than in the Canadian study (≥ 12 months). Communication and cognitive problems subunit scores were close to each other in both studies, suggesting that patient perceptions are similar concerning the treatment’s long-term effects. The perceived physical appearance and anxiety subunit scores are also higher in the Canadian study, which can be due to the differences in socioeconomic, developmental, and cultural level issues and access to health services in both countries (Table 3).

**Table 3:** Comparison of QL with other studies.

	Sung et al.	Tsuji et al. child	Tsuji et al. proxy	Varni et al. child	Varni et al. proxy	Abu Saad et al. child	Our study child report	Our study proxy report
Mean of total score		79.4	74.3			77.8	75.6	69.4
Pain and hurt	62.5	86.2	81	76.2	74.7	85.3	82.5	78.9
Nausea	70	83.8	82.9	75.8	77.8	76.2	82.9	76.6
Procedural anxiety	51.2	78.2	68.6	68.3	60.3	92.3	65.7	62.4
Treatment anxiety	75	94.6	87.2	82.2	71.5	90.2	82.4	66.4
Anxiety	100	78.8	79	70.1	75.9	79.1	79.0	68.3
Perceived physical apperarance	91.7	72	69.1	70.3	76.2	92.1	69.1	70.2
Communication	75	66	60.9	74.4	78.3	86.9	72.1	65.7
Cognitive problems	65	71.4	64.8	70.5	74	88.7	71.7	67.1

In our study, the sex differences only appear in the proxy report’s perceived physical appearance subunit. Lower physical appearance scores were found in male patients’ parents, which is just the opposite of the finding in the study of Vlachioti et al. [14]. The reason for lower scores in parents of male patients in our study may be the limited ability to cope with male patients’ physical changes compared to female patients. Following our study, Tsuji et al. [17] found higher total QL scores in leukemic children than their parents’ proxy reports. Higher subunit scores and the lowest subunit scores were shown in Table 3. In accordance with the study of Varni [5], proxy reports showed significant improvements only in treatment anxiety and procedural anxiety subunits. Considering the QL evaluations of children and parents, the study by Tsuji et al. [17] and Varni et al. [5] seem to be similar to our study (Table 3). 

There were no improvements in the subunit scores that are considered as long-term effects [18] like perceived physical appearance and cognitive problems in the studies of Abu Saad et al. [18] and Varni et al. [5]. and our research. Psychosocial issues originate from the recurrence anxiety of cancer that could be at an unconscious level in children. Besides, emerging adverse events during treatment and long-term side effects of the therapy also cause stress, leading to low QL subunit scores [18,19] (Tables 2 and 3).

Health-related QL scores were significantly lower in a study from Norway consisting of childhood ALL survivors with a mean age of 11.8 years. Although cognitive functions were within normal limits, their scores were lower than those of healthy children [4]. 

Higher RPM scores of female patients in the second and fifth grades could be due to the increased parental educational level and the support given by these educated parents. Unfortunately, the case number is too small to perform a regression analysis to find where this difference arises. 

It was also reported that neurocognitive deficits could still be seen even five years or more after completing treatment [20]. Reading, writing, and mathematics skills were associated with the off-treatmentt time in our study.

Inconsistent with the study of Kunin-Batson et al. [21], 40% of our patients had writing and mathematical difficulties, and 60% had reading difficulties. 

Learning difficulties in every field cause academic life failure and indirectly low QL and dissatisfaction in the future. Other studies in this area also show that patients have long-term side effects like poor school performance, decreased job finding, and poor mental health, which designates that QL is negatively affected [20,21]. These side effects are related to the psychological trauma due to a life-threatening disease, staying in the hospital for a long time, leaving the family and friends [20,21]. 

In our study, parental education level and family income positively influence reading and mathematics skills. It is thought that patients with supportive families have less difficulty when they return to school.

Intensive and prolonged treatment protocols in childhood cancers lead to immobility and lower physical performance levels. However, it has been shown that children with cancer should be physically active and participate in an individually tailored exercise program [22]. Depending on the affected physical functions, various social isolation problems, loss of independence, anxiety, and depression might be seen. These physical and psychosocial changes negatively affect the QL of both the family and the children [22–24]. Our study showed that the patient’s physical independence has recovered as the time elapsed over the intensive treatment that also contributes to social interaction and daily independence increases. Parent-and-child assessments of QL scores improve.

There was no significant difference in gross motor skills than the control group; there was significant retardation in our study’s fine motor skills. Our patients’ age was within fine motor skills development, where chemotherapy and hospitalization could have hampered this process.

It has been reported that fine motor skills difficulties increase after treatment, and handwriting problems last almost two years after treatment in approximately 25% of children [22–25]. Inconsistent with Hartman et al. [24] study though motor performance improves, it is still lower than healthy peers even two years after cessation of treatment. Although some subunits of gross motor skills improved with remission, our patients’ BOTMP SF scores did not reach standard children’s’ BOTMP SF scores. 

Even though our study population seems a small national cohort, many patients from different geographic areas were referred to our center. We recruited all our patients in our registry, which required inclusion criteria. As an experienced pediatric leukemia center in a developing country, we aimed to reveal the contributors to the quality of life after the end of the therapy. These evaluations from different aspects of the child’s development were performed at once. Unfortunately, the patients could not be evaluated at the same period after the therapy due to our social conditions. Thus, the results compared according to average values align with the age and the mean duration after the remission. All patients were investigated according to the mean off- treatment time. Future research with routine follow up of QL outcomes using multidisciplinary measures during treatment and off-treatment will be crucial.

## 5. Conclusion

Our study using disease-specific QL in children with ALL in remission revealed three significant findings.

1. There was no effect of off-treatment time on pain, anxiety, cognitive problems, perceived physical appearance, and the total QL scores of children and proxy reports.

2. Children’s physical capacities were significantly worse than healthy controls and have not reached the level of healthy children even after a long time since the end of the therapy.

3. Reading, writing, and mathematical skills were significantly associated with off-treatment time. The total score of RPM and PedsQL of those with mathematical difficulties were significantly lower than those without any difficulty.

Our study also suggests that the inclusion of physical activity in developing a cancer-specific scale may provide a more precise assessment. As motor skills difficulties will lead to low academic recognition could direct the parents for immediate intervention. Children and their families should be supported to participate in these programs to ensure progress both in social communication and academic achievement. 

## Informed consent

Informed consent and basic demographic information by face to face interview, PedsQL 3.0 child and proxy report, for ages 5–7 years and 8–12 years (5), WeeFIM scale (6), BOTMP-SF (7), RPM, reading, writing, mathematics assessment tool (8,9) were carried out children and their families (Supplementary material). This study was approved by the Gazi University Faculty of Medicine Ethics committee (#2016-2/15).
